# Supplementary material for: Mosaic: in-memory computing and routing for small-world spike-based neuromorphic systems
Source: Nat Commun. 2024 Jan 2;15:142. doi: 10.1038/s41467-023-44365-x (PMC10761708; doi:10.1038/s41467-023-44365-x)
Supplement: Supplementary file 1 — Supplementary information [file 41467_2023_44365_MOESM1_ESM.pdf]

# Supplemental Materials:
# Mosaic: in-memory computing and routing for small-world spike-based neuromorphic systems

## Supplementary Note 1

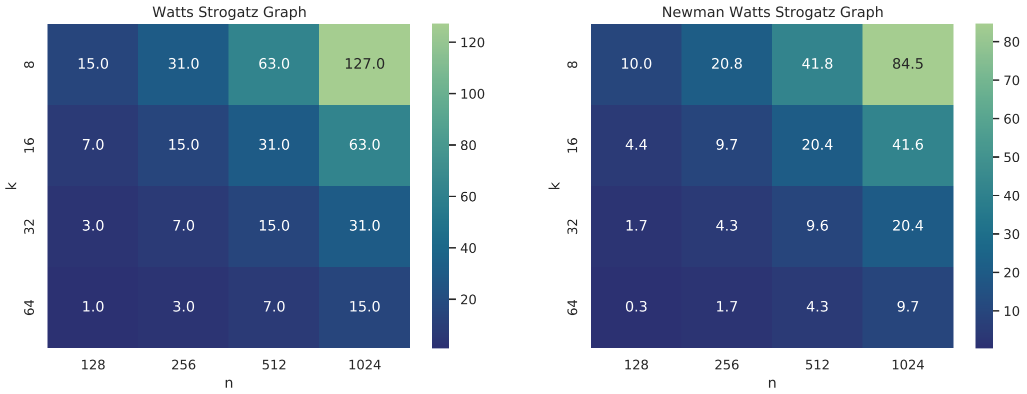


Figure S1: The heatmaps show the ratio of zero elements to non-zero elements in the connectivity matrix for two examples of recurrently connected small-world graph generators. As $n$ (number of nodes, e.g., neurons, in the graph) increases and $k$ (number of neighbour nodes in a ring topology, each node is connected to) decreases, the more connections in the connectivity matrix will be zero, indicating the increased proportion of non-used memory elements in a $n \times n$ crossbar array.

Figure S1 quantifies the under-utilization of conventional crossbar arrays while storing example small-world connectivity patterns generated by two standard random graph generation models: Watts-Strogatz small-world graphs [1] and Newman-Watts-Strogatz small-world graphs [2]. The first type of graphs is characterized by a high degree of local clustering with short vertex–vertex distances, observed in neural networks and self-organizing systems, whereas the latter type mostly captures the properties of lattices with which statistical physics deals with.

## Supplementary Note 2

To communicate the events between the computing nodes in neuromorphic chips, Address-Event Representation (AER) communication scheme has been developed and used [3]. In AER, whenever a spiking neuron in a chip (or module) generates a spike, its “address” (or any given ID) is written on a high-speed digital bus and sent to the receiving neuron(s) in one (or more) receiver module(s). In general, AER processing modules require at least one AER input port and one AER output port. As neuromorphic systems scale up in size, complexity, and functionality, researchers have been developing more complex and smarter AER “variations” to maintain the efficiency, reconfigurability, and reliability of the ever-growing target systems they want to build. The scheme that is used to transport events can be source or destination based, where the source or destination address is embedded in the sent event “packet”. In the source-based scheme, each receiving neuron has a local Content Addressable Memory (CAM) that stores the address of all the neurons that are connected to it. In the destination-based approach, each event hops between the nodes where its address gets compared to the node’s address until it matches and thus gets delivered. Source-driven routing provides the designer with more freedom to balance event traffic and design routes, but the hardware complexity increases the delays. Destination-based creates pre-determined routes along the network and the designer can only change the output ports [4]. In summary, in source-based routing, the system requires a CAM memory per neuron, which results in an increase in the area and memory access read times. In destination-based routing, the configurability in the network structure is reduced. Comparatively, in the Mosaic, the routers are memory crossbars that are distributed between the computing cores and steer the spiking information in the mesh.

## Supplementary Note 3

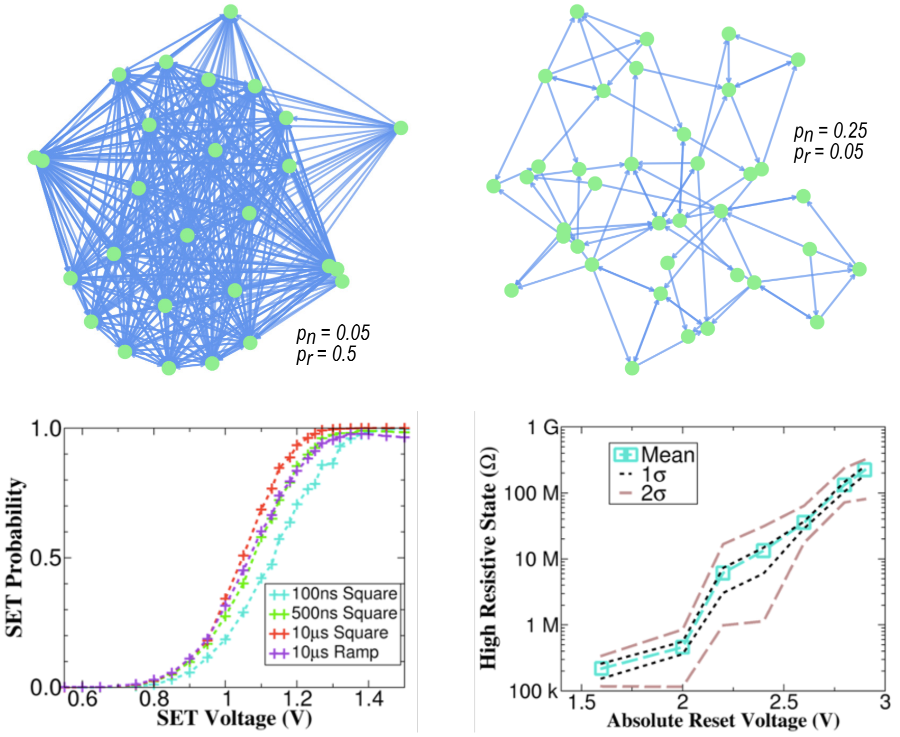


Figure S2: (top) Different random graphs generated using Mosaic model, changing the probability of devices being in their High Conductive state in the neuron tile ($p_n$) and routing tile ($p_r$). (bottom) The probability of device switching is a function of the voltage applied to it while being programmed.

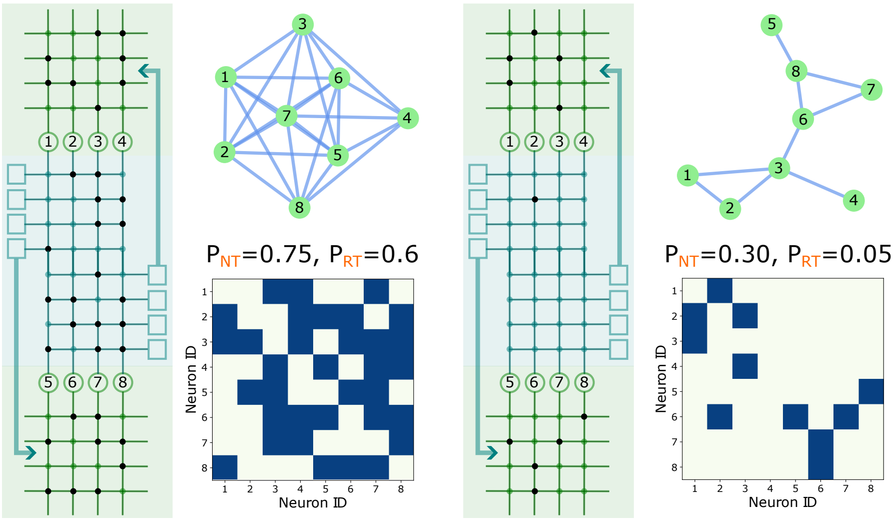


Figure S3: Mosaic connectivity example, formed by setting the probability of connection within Neuron Tile ($p_{NT}$) and Routing Tiles ($p_{RT}$). (left) Densely connected Mosaic composed of 2 Neuron Tiles and 1 Routing Tile. The graph related to its connectivity is shown as well adjacency matrix. (right) Sparsely connected Mosaic. The graph is programmed to favor the intra-Neuton Tile connectivity and allow for two clusters to emerge, penalizing connections between the two clusters.

Routing tiles define the connectivity of spiking neural networks implemented on Mosaic. When the number of memristive devices in the routing tiles which are in their high-conductive state (HCS) is not sparse, Mosaic resembles a densely connected neural network (Fig. S2, top left). When most of the memristor in the routing tiles are in the low-conductance state, Mosaic is sparsely connected (Fig. S2, top right). Furthermore, one can further sparsify Mosaic networks by setting memristors in the neuron tiles to the LCS. To do so, we can change the probability of memristors being in their HCS in the neuron tiles, $p_n$, and in the routing tiles, $p_r$. The

switching of the Resistive Random Access Memorys (RRAMs) presents the property of probabilistic switching as a function of the voltage applied during the programming operation as is shown in Fig. S2, bottom.

Fig. S3 shows the construction of two graph topologies, made of 2 Neuron Tiles and one Routing Tile, to clarify the formation of the graphical structure in the Mosaic. By controlling the probability of connections within the Neuron and Routing Tiles, we can produce a densely connected graph (left) with $p_{NT} = 0.75$, $p_{RT} = 0.6$, and a sparse graph (right) with $p_{NT} = 0.30$, $p_{RT} = 0.05$.

The corresponding connectivity matrix is also shown in the figure, which is directly represented as a hardware architecture in the 3 tiles of the Mosaic, as shown in the figure.

## Supplementary Note 4

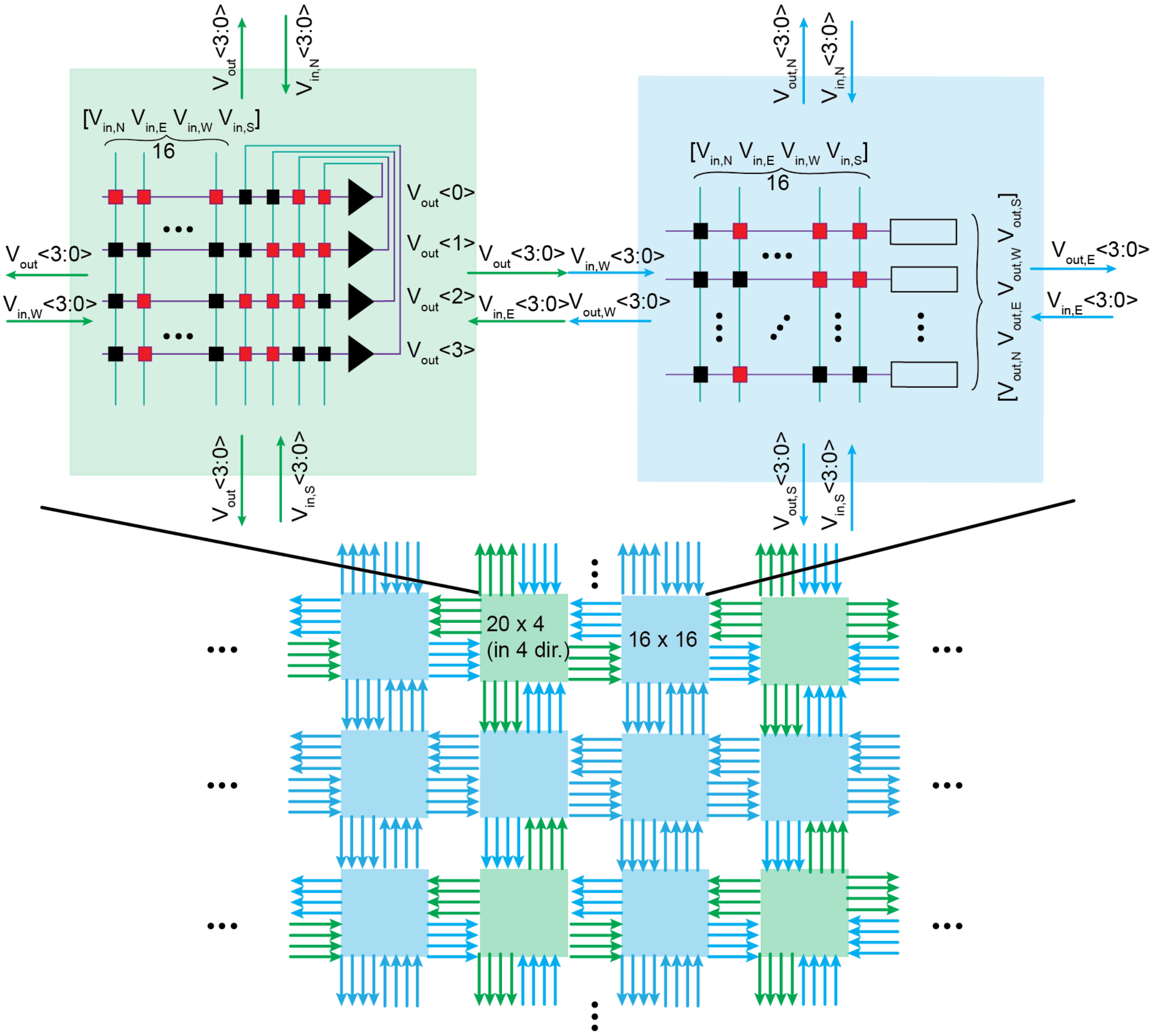


Figure S4: (Neuron tiles (green) transfer information in the form of spikes to each other through routing tiles (blue). Details of the Mosaic architecture is shown with the size of the neuron and routing tiles. The neuron tiles receive feed-forward input from four directions of North (N), East (E), West (W), and South (S), and local recurrent input from the neurons in the tile. The neurons integrate the information and once spike, send their output to 4 directions. Having 4 neurons in a tile, gives rise to 16 outputs (4 outputs copied in 4 directions), and 20 inputs (4 inputs from 4 directions (16), plus 4 recurrent inputs). The routing tiles receive 16 inputs (4 inputs from 4 directions) and send out 16 outputs (4 outputs in 4 directions). In the crossbars, the red squares and black squares represent devices in their high conductive and low conductive state, respectively. The connection between the neuron tile and the routing tile is directly through a wire. For instance, $V_{\text{out}} < 3{:}0 >$ is the same as the $V_{in,W}$, and $V_{\text{in,E}} < 3{:}0 >$ is the same as $V_{out,W}$.

Figure S4 shows the details of the Mosaic architecture, with a zoomed in neuron and routing tile pair. The diagram in the top shows how one cluster of neuron/one router sends and receives information to and from the routing/neuron tile. This highlights the strength of this architecture which makes the connectivity easy through simple wiring to the neighbour, without suffering from long wires, as the maximum length of a wire is the size of the wire from one row/column, plus the size of the connecting column/row.

## Supplementary Note 5

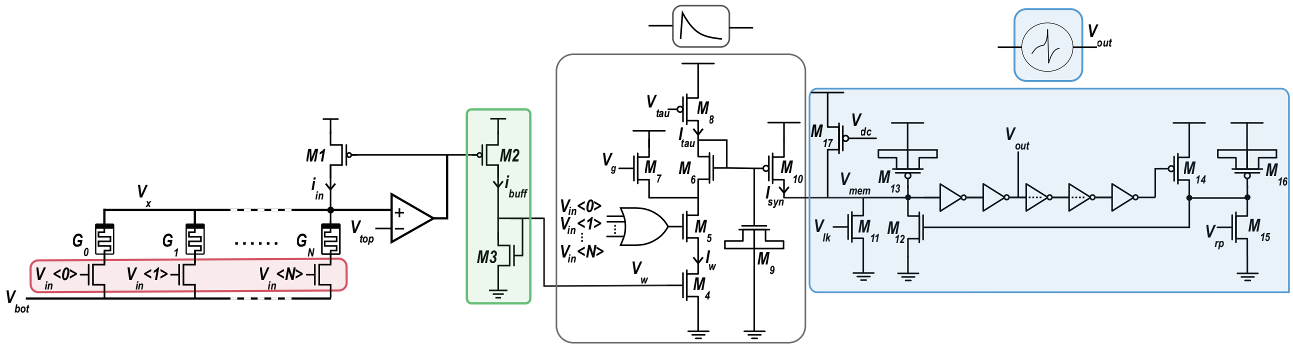


Figure S5: Schematic of the neuron tile including the CMOS synapse and neuron circuits fabricated for use in this paper. RRAMs are used as the weights of the neurons. On the arrival of any of the input events $V_{in<i>}$, the amplifier pins node $V_x$ to $V_{top}$ and thus a read voltage equivalent to $V_{top} - V_{bot}$ is applied across $G_i$, giving rise to current $i_{in}$ at $M_1$. This current is mirrored to $M_2$ giving rise to $i_{buff}$ which is in turn again mirrored through the $M_3 - M_4$ transistor pair. The "synaptic dynamics" circuit is the Differential Pair Integrator (DPI) [5]. On the arrival of any of the input events, $V_i, 0 < i < n$, $I_w$, equivalent to $i_{buff}$, flows in transistor $M5$. Depending on the value on $V_g$, a portion of $I_w$ flows out of the MOS capacitor M6 and discharges it. This current is proportional to $G_i, 0 < i < n$. As soon as the event is gone, MOS capacitor $M_6$ charges back through the $M_8$ path with current $I_{tau}$, which determines the rate of charging, and thus the time constant of the synaptic dynamics. The output current of the DPI synapse, $I_{syn}$, is injected into the neuron's membrane potential node, $V_{mem}$, and charges MOS capacitor $M_{13}$. There is also an alternative path with a DC current input through $M_{17}$ which can charge neuron's membrane potential. Membrane potential charging has a time constant determined by $V_{lk}$ at the gate of $M_{11}$. As soon as the voltage developed on $V_{mem}$ passes the threshold of the following inverter stage, it generates a pulse. The width of the pulse, depends on the delay of the feedback path from $V_{out}$ to the gate of $M_{12}$. This delay is determined by the inverter delays, and the refractory time constant. The inverter symbols with the horizontal dashed lines correspond to a starved inverter circuits with longer delays. The refractory period time constant depends on the MOS cap $M_{16}$ and the bias on $V_{rp}$.

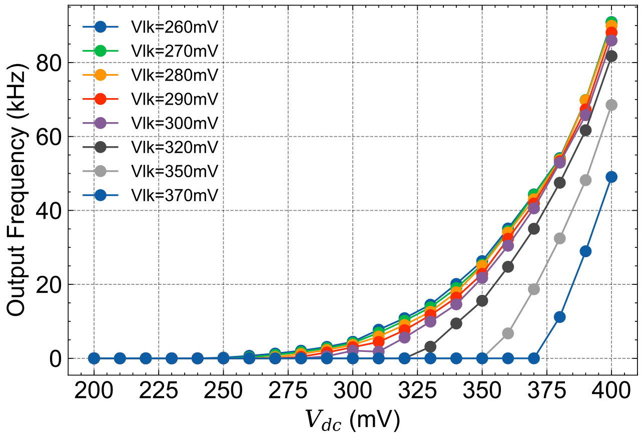


Figure S6: Measurements from fabricated neuron's output frequency as a function of the input DC voltage. The DC voltage is applied at the gate of transistor $M_{17}$ shown in Fig. S5 as $V_{dc}$. Therefore, as the gate voltage of $M_{17}$ changes linearly, the current of $M_{17}$ and thus the output frequency of the neuron changes non-linearly. Each curve is measured with a different neuron's time constant, determined by a different voltage, $V_{lk}$, on the gate of transistor $M_{11}$ in Fig. S5. As the leak voltage increases, the neuron's time constant decreases, giving rise to a lower output frequency.

Details of the implementation of the neuron row, the circuit that leverages the information of the conductance of a memristor to weight the effect of a spike to a neuron is shown in Figure S5. The circuit features multiple inputs connected to a row of memristive devices (left) and a Front-End circuit buffering the current read from the devices to a differential-pair-integrator synapse. The synapse is then connected to a leaky-integrated-and-fire (LIF) neuron which eventually emits a spike. Figure S6 delves deeper in the behavior of the LIF neuron analyzing its output spiking frequency against an input DC voltage and its linear behavior respect to the RRAM conductance in a neuron row circuit.

## Supplementary Note 6

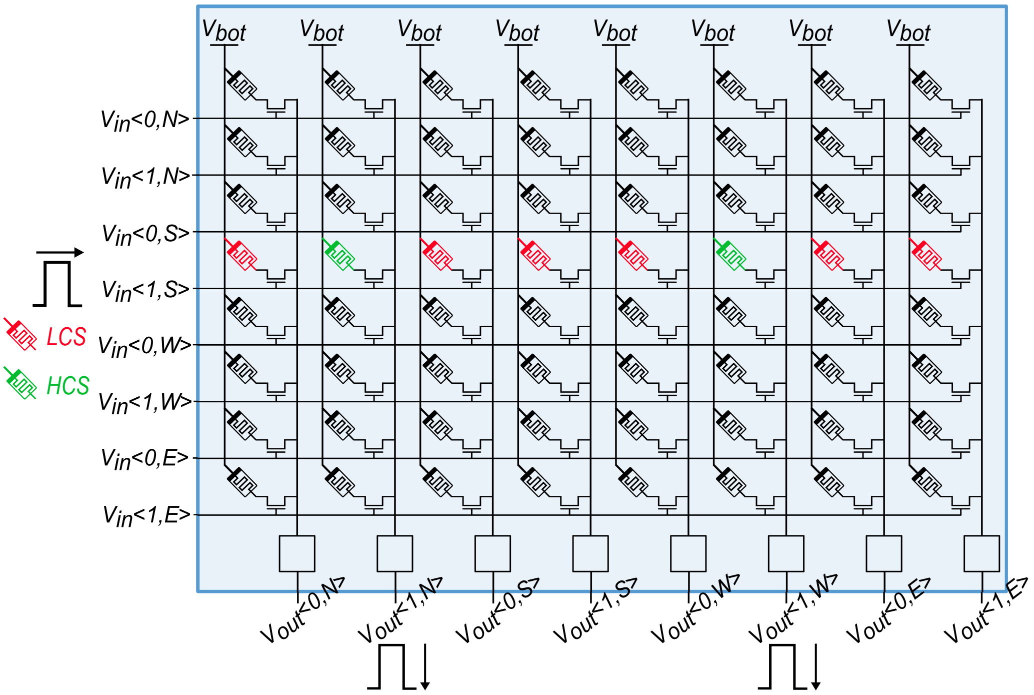


Figure S7: Schematic of a routing tile circuit offering two paths per direction. The routing tile receives eight inputs, comprising two pulse channels per direction, labelled as $< 0 >$ or $< 1 >$, from the neighbouring tiles to the North (N), South (S), East (E) and West (W), and provides complimentary outputs. An example is shown of an input pulse arriving to the common gate of the fourth row of memory. Devices are coloured green or red to denote whether they are in the High-Conductive State (HCS) or Low-Conductive State (LCS). It is shown that, due to this input pulse, output pulses are produced by the routing columns containing the (green) devices programmed in the HCS.

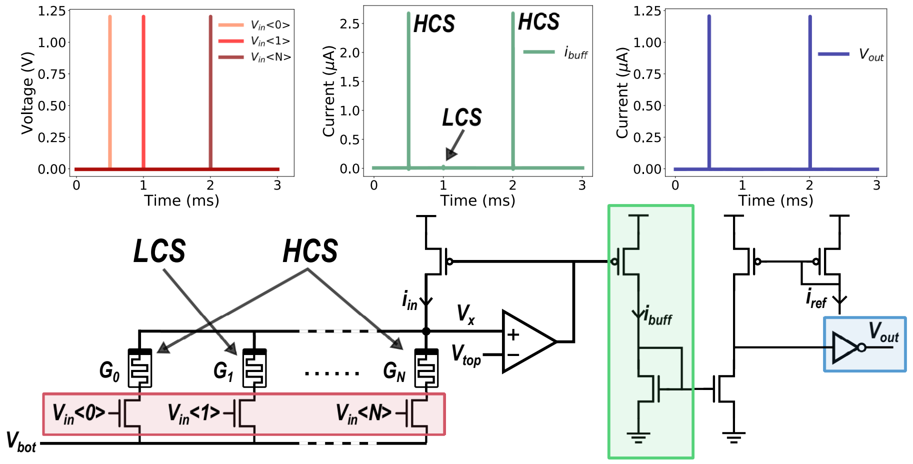


Figure S8: The routing column circuit with example waveforms. Input (red, left) voltage pulses, Vin, draw a current iin proportional to the conductance state, Gn, of the read 1T1R structures. Two devices are labelled with HCS, indicating that they have been programmed with a conductance corresponding to the high conductance state, and one is labelled LCS in reference to the low conductance state. This resulting currents are buffered (green, centre), ibuff , into a current comparator circuit where it is compared with a reference current iref . When the buffered current exceeds the reference current a voltage pulse is generated at the column output (blue, right).

Details on the implementation of the Routing Tiles. Figure S7 shows a full-size schematics of a routing tile with 2 neurons allocated per direction. Figure S8 expands on the details of the implementation of the routing column, the circuit that uses the state of a memristor to decide whether to block or pass (route) a spike through the Mosaic architecture.

## Supplementary Note 7

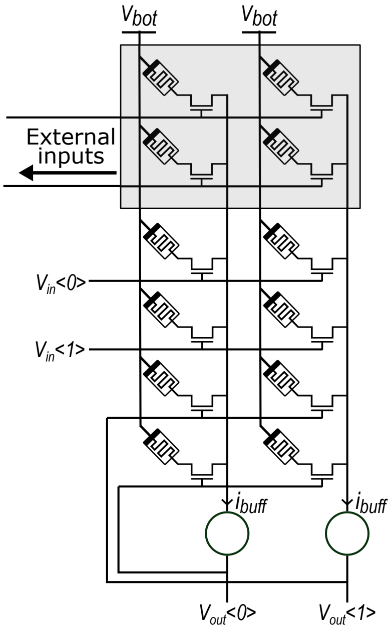


Figure S9: An example of how a neuron tile can be interfaced to external event-based inputs (i.e., those generated by an event-based sensor). With respect to the neuron tile circuit presented in the paper (permitting connections to adjacent tiles as well as recurrent connections within the tile), this figure shows two additional rows of devices stacked on top of the array. As an arbitrary example, here two additional signals can be integrated in the neuron circuits.

## Supplementary Note 8

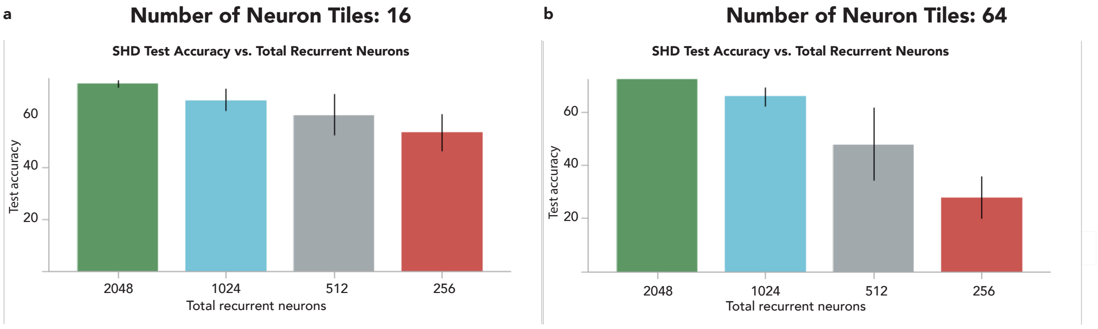


Figure S10: SHD keyword spotting dataset test accuracies for Mosaic architectures with different total number of neurons in the network for a) 4x4 Neuron Tile layout (a total of 16 number of Neuron tiles) and b) 8x8 Neuron Tile layout. The number of neurons per tile is equal to the total number of recurrent neurons divided by the number of neuron tiles. Median and standard deviation are calculated using 3 experiments with varying sparsity constraints.

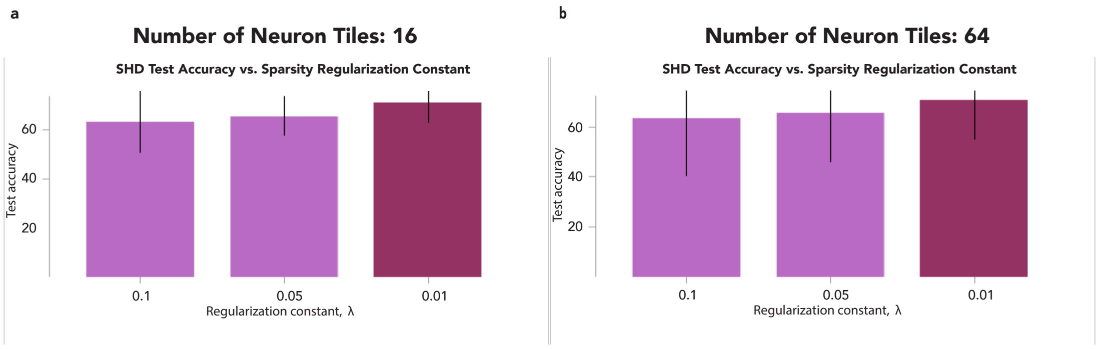


Figure S11: SHD keyword spotting dataset test accuracies for Mosaic architectures trained with different sparsity regularization values. As explained in the Methods section of the main text, the regularization is added to the loss function to exponentially penalize the long-range connections. The plot shows the accuracy for strong (default, $\lambda = 0.1$), medium ($\lambda = 0.05$) and weaker ($\lambda = 0.01$) sparsity regularization on a) 4x4 neuron tile layout and b) 8x8 neuron tile layout. Median and standard deviation are calculated using 4 experiments with varying number of neurons per neuron tile.

## Supplementary Note 9

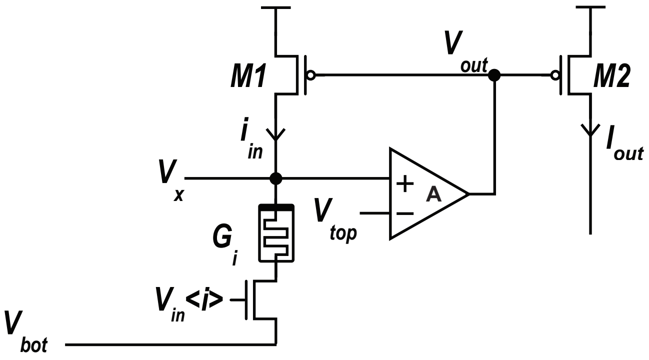


Figure S12: Analysis of the read-out circuitry. The amplifier with gain A, pins voltage $V_x$ to the voltage $V_{top}$. On the arrival of the pulse on $V_{in} < i >$, a current equal to $i_{in} = (V_{top} - V_{bot})G_i$ flows into the memristor $i$, which is then mirrored out $i_{out}$.

Figure S12 details the implementation of the read-out circuit used in the Mosaic architecture. Though not optimized for area, we have used this implementation for both the neuron and routing tiles.

The dominant power consumption of the circuit depends on the required bandwidth (BW) of the feedback loop. This BW depends on the maximum conductance of the RRAM, $G_{max}$. For $G_{i,max}$, once an input arrives to $V_{in} < i >$, the current $i_{in}$ has to settle to $(V_{top} - V_{bot})G_{i,max}$ within a settling time, $t_s$, a proportion of the pulse width. This timing sets the speed at which the loop should work, and thus its BW. If the loop does not close in this time, the amplifier will slew, and the voltage $V_x$ drops. In both neuron and routing tiles, this condition should be met for $V_x$ to stay pinned at $V_{top}$ while RRAM is being read. However, the neuron and routing tiles have different BW requirements.

In the **neuron tile**, the read-out circuitry has to resolve between at least 8 levels of current for the 8 levels that each RRAM device can take. Therefore, the Least Significant Bit (LSB) of the $i_{in}$ current for the neuron tile is $i_{in,LSB,N} = \frac{V_{ref}(G_{max}-G_{min})}{N}$. Based on the Fig. 2d, this value for the neuron tile is $\frac{100mV(120\mu S-40\mu S)}{8} = 1\mu A$. Note that since the 8 levels to be resolved are in the Low Resistive State of the RRAM, the $G_{max}$ and $G_{min}$ are the minimum and maximum of the range in the LRS, which correspond to $40\mu S$ and $120\mu S$.

In the **routing tile**, the read-out circuitry has to resolve between two levels which will either let the spike regenerate and thus propagate, or will get blocked. Therefore, the LSB of the $i_{in}$ current in the routing tile is $i_{in,LSB,R} = \frac{V_{ref}(G_{max}-G_{min})}{N}$. Based on the Fig. 2d, this value for the neuron tile is $\frac{100mV(40\mu S-10\mu S)}{2} = 15\mu A$. Note that since the 2 levels to be resolved are the LRS and HRS of the RRAM, the $G_{max}$ and $G_{min}$ correspond to $10\mu S$ and $40\mu S$.

To be able to distinguish between any two levels in both cases, we will consider a maximum error of $\frac{i_{in,LSB}}{2}$. Therefore, the maximum tolerable error in the the neuron tile is $0.5\mu A$ and in the routing tile is $7.5\mu A$.

This means that if the feedback loop does not close in $t_s$ of the pulse width, $V_x$ drop is a lot more tolerable in the routing tile than it is in the neuron tile. This suggests that the bandwidth requirements in the case of

neuron tile is $7.5/0.5 = 15$ times more than that of the routing tile. The BW requirements directly translate to the biasing of the amplifier and thus its power consumption. Therefore, the static power consumption of the neuron tile is 15 times that of the routing tile. The current requirements also translate to area, since larger currents require wider transistors.

# References


[1] Duncan J Watts and Steven H Strogatz. Collective dynamics of ‘small-world’networks. *Nature*, 393(6684):440–442, 1998.

[2] M.E.J. Newman and D.J. Watts. Renormalization group analysis of the small-world network model. *Physics Letters A*, 263(4-6):341–346, December 1999.

[3] K. Boahen, M. Nomura, E. Ros Vidal, and R. Van Rullen. Address-event senders and receivers: Implementing direction-selectivity and orientation-tuning, 1998.

[4] Carlos Zamarreño-Ramos, L.A. Camuñas-Mesa, J.A. Pérez-Carrasco, Timothée Masquelier, Teresa Serrano-Gotarredona, and Bernabé Linares-Barranco. On spike-timing-dependent-plasticity, memristive devices, and building a self-learning visual cortex. *Frontiers in Neuroscience*, 5:1–22, 2011.

[5] Chiara Bartolozzi and Giacomo Indiveri. Synaptic dynamics in analog vlsi. *Neural computation*, 19(10):2581–2603, 2007.
